# Prevalence of the *CHEK2* R95* germline mutation

**DOI:** 10.1186/s13053-016-0059-0

**Published:** 2016-09-27

**Authors:** Stian Knappskog, Beryl Leirvaag, Liv B. Gansmo, Pål Romundstad, Kristian Hveem, Lars Vatten, Per E. Lønning

**Affiliations:** 1 Section of Oncology, Department of Clinical Science, University of Bergen, 5020 Bergen, Norway; 2 Department of Oncology, Haukeland University Hospital, Bergen, Norway; 3 Department of Public Health, Faculty of Medicine, Norwegian University of Science and Technology, Trondheim, Norway

## Abstract

**Background:**

While germline *CHEK2* mutations have been linked to a moderately elevated cancer risk, to date, a limited number of such mutations have been identified. Recently, we reported a germline nonsense mutation (C283T; R95*), introducing an early stop-codon, in two Norwegian patients diagnosed with locally advanced breast cancer. Both patients were resistant to anthracycline therapy, resembling what has been observed for *TP53* mutations.

**Methods:**

In the present study, we screened a large population based sample, including 3748 non-cancer individuals and 7081 incident cancer cases (breast cancer, *n* = 1717; prostate cancer *n* = 2501, lung cancer *n* = 1331 and colorectal cancer *n* = 1532), for the distribution of *CHEK2* R95*.

**Results:**

We found that 12 individuals (0.11 %) carried the R95* variant: 4 non-cancer individuals (0.11 %), 4 breast cancer cases (0.23 %), and 4 prostate cancer cases (0.16 %). Although the low number of observations precluded formal statistical assessment, our data may indicate an elevated risk for breast (OR: 2.19, 95 % CI: 0.55–8.75) and prostate cancer (OR: 1.5, 95 % CI: 0.36–6.00) associated with *CHEK2* R95*. By mining international databanks, we found no individuals carrying the R95* mutation, indicating it to be restricted to the Norwegian population.

**Conclusion:**

We provide proof-of-concept that previously unknown *CHEK2* germline mutations may be present in certain populations. Notably, germline mutations in tumours are in general missed by contemporary massive parallel sequencing strategies, since tumour mutations are usually filtered against the germline. The fact that the *CHEK2* R95* mutation may be associated with resistance to anthracyclines in cancer patients emphasizes its possible clinical importance.

## Background

Checkpoint kinase 2 (CHEK2) has a critical role as a tumour suppressor, activating p53 in response to genotoxic stress. A limited number of germline point mutations in *CHEK2* have been identified. In general, these are present at low frequencies and have moderate impact on cancer risk: the most frequent and most studied variant, the 1100delC truncation, occurs in approximately 1 % of Northern Europeans [1–3]. Unlike single amino acid substitutions, the 1100delC truncation variant has been associated with an increased risk for cancer of the breast and prostate [1, 3]. Also, a larger 5395 bp germline deletion observed in individuals of Eastern European descent, has been linked to increased risk of breast cancer [1, 4, 5], and recently several germline truncating *CHEK2* mutations were linked to risk of Non-Hodgkin Lymphoma [6]. Taken together, these, and other data clearly indicates *CHEK2* to be a multi-cancer susceptibility gene, with truncating mutation being of particular importance for some cancer types [7].

**Fig. 1:**
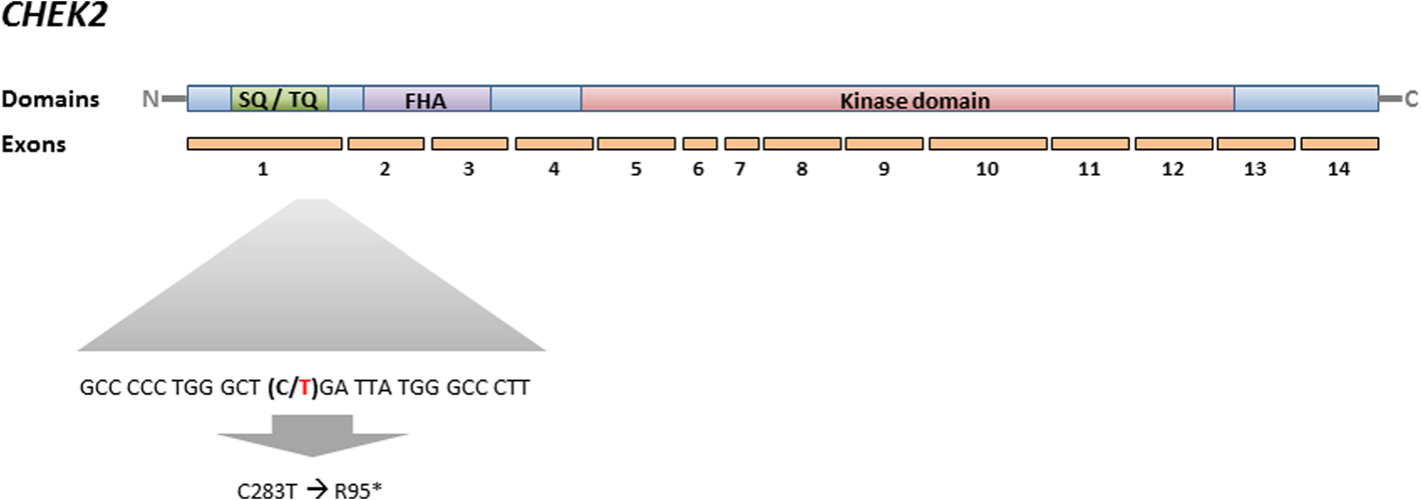
Schematic representation of the *CHEK2* gene with details on the flanking region of the R95* mutation (in bold; C = reference allele, T = alternative allele)

In a previous study, we found two unrelated patients to harbour a germline C – T transition in position 283 of the coding region of *CHEK2* (rs587781269; Fig. 1). This transition introduced a novel translation stop at codon 95 (CGA → TGA; R95*) causing a severely truncated protein [8]. The mutant protein proved to be non-functional both in terms of kinase activity and dimerization in in vitro assays [8]. Importantly, both patients carrying the R95* variant revealed primary resistance to anthracycline therapy [8], resembling what we observed for patients with tumours harbouring somatic *TP53* mutations in the same study [8–10].

The aim of the present study was to determine the *CHEK2* R95* mutation incidence and potential influence on cancer risk across the general population.

## Methods

### Study population

All samples (DNA from blood) analyzed (*n* = 10,830) were obtained from the population-based Cohort of Norway (CONOR) study [11]. Participants were from all regions of Norway, but the majority (*n* = 4131) were residents of Nord-Trøndelag County in Mid-Norway. The mean age at sampling was 61.2 years (range 20.0–93.6 years). Incident cancers were identified by linking the identity of individuals to the Norwegian Cancer Registry. We analyzed 1717 incident cases of breast cancer, 1532 colon cancers, 1331 lung cancers and 2501 prostate cancers, in addition to 3749 control individuals without cancer. The 10,830 individuals are identical to the sample set previously described [12]. One sample failed analysis, thus, samples from 10,829 individuals were used for data assessments. All sample donors provided written informed consent to anonymous genetic testing for scientific purposes, and the study was approved by the Regional Committee for Ethics in Medical Research (REK Midt-Norge).

### *CHEK2* R95* genotyping

All samples were genotyped for the *CHEK2* R95* mutation using a custom LightSNiP assay (TIB MOLBIOL Syntheselabor GmbH, Berlin, Germany) on a LightCycler 480 II instrument (Roche, Basel, Switzerland). Reaction mixes and thermocycling conditions were set according to the manufacturer’s instructions

## Results

In order to assess the frequency of the *CHEK2* R95* mutation in the general Norwegian population, we genotyped peripheral blood DNA from a previously described [12] sample set (*n* = 10,829) extracted from the population based CONOR biobank. The *CHEK2* R95* mutation was observed in four out of 3748 individuals without any cancer diagnosis (0.11 %; Minor allele frequency [MAF] 5.3 × 10^−4^; Table 1), in four out of 1717 breast cancer patients (0.23 %), and in four out of 2501 prostate cancer patients (0.16 %). In contrast, none out of 1331 lung cancer and none among 1532 colorectal cancer patients harboured the mutation (Table 1). While the low number of observations precluded any formal statistical assessment of the role of R95* as a potential cancer risk factor, our data may indicate an elevated risk for breast (OR: 2.19, 95 % CI: 0.55–8.75) and prostate cancer (OR: 1.5, 95 % CI: 0.36–6.00) among mutation carriers. Supporting this, we found the age at diagnosis among the breast and prostate cancer patients to be slightly lower among the R95* carriers than among the *CHEK2* wild-type patients (Table 2).

**Table 1:** Frequency of the *CHEK2* R95* germline mutation in the Norwegian population

Individuals/patients	R95*-carriers^a^ *n*	Wild-type *n*	Total	MAF
Non-cancer individuals	4	3744	3748	5.3 × 10^−4^
Cancer patients	8	7073	7081	5.6 × 10^−4^
Breast cancer	4	1713	1717	1.2 × 10^−3^
Prostate cancer	4	2497	2501	8.0 × 10^−4^
Lung cancer	0	1331	1331	0
Colorectal cancer	0	1532	1532	0
Total	12	10,817	10,829	5.5 × 10^−4^

^a^All R95* carriers were heterozygous

**Table 2:** Age at cancer diagnosis

	Average age at cancer diagnosis		
Diagnosis	R95*-carriers	Wild-type	Total
Breast cancer	55.75	60.36	60.35
Prostate cancer	69.75	71.72	71.72

We performed data mining in order to assess whether the R95* mutation was restricted to certain geographical areas: We searched the 1000 genomes project phase 3, providing genomic data for 2439 individuals of different ethnic origin including several Caucasian sub-populations [13]: none of them harboured the R95* mutation, indicating that the variant may be restricted to the Norwegian population. The CONOR biobank contains samples collected from individuals living in distinct parts of Norway; thus, some regions are represented in particular. Accessing information about the county of residence for each individual donor, we found 11 out of 12 individuals harbouring the R95* mutation to be residents of the Nord-Trøndelag County in Mid-Norway (consisting of about 2.6 % of the Norwegian population and contributing 4131 individuals to our cohort), contrasting one R95* mutation carrier out of 6698 participants living in other parts of Norway, (difference in incidence: *p* = 1.9 × 10^−4^). Restricting the association estimates to residents of Nord-Trøndelag did not have any major impact on the results (OR: 1.49, 95 % CI: 0.33–6.66 for breast cancer and OR: 1.32, 95 % CI: 0.33–5.30 for prostate cancer).

## Discussion

In the present study, we screened a large population based cohort, and found the prevalence of the *CHEK2* R95* mutation to be low and restricted to Norway.

Despite the low frequencies of the individual mutations, precluding proper statistical assessment of the odd ratios, germline *CHEK2* mutations seem to be the underlying cause of hereditable cancer syndromes in some families [1, 2, 14]. To date, the most studied *CHEK2* germline mutation is the 1100delC variant, which has a relatively high frequency in the Caucasian population: approximately 1 % of healthy individuals are estimated to carry the 1100delC-variant [1–3]. Contrasting the risk assessments of single amino acid substitutions of the CHEK2, it has been estimated that the 1100delC-variant confers a two-fold elevated risk of both breast cancer [3] and prostate cancer [1]. Further, another deletion variant in Eastern Europe (5395 bp deletion) has also been linked to increased breast cancer risk [1, 4, 5] and several germline truncating variants have been linked to Non-Hodgkin lymphoma [6].

Recently, another novel germline *CHEK2* mutation, Y390C, was reported [15]. Interestingly, this mutation was associated with elevated risk for breast cancer but also resistance towards anthracyclines in vitro. In an earlier study, we also reported another truncating *CHEK2* mutation, (ins1368A), to be associated with resistance to anthracycline therapy in a breast cancer patient [16]; thus, all these three mutations (R95*, Y390C and ins1368A) seem associated with defect DNA damage signaling leading to drug resistance. Notably, this contrasts some of the previously reported missense mutations (e.g. I157T and I364T) that seem to retain a wild-type kinase activity [8]. While we may not draw any final conclusion due to a limited number of observations, our data are consistent with the hypothesis [1–5] that certain *CHEK2* mutations (as opposed to most missense mutations) may be associated with elevated cancer risk and, more importantly, resistance towards DNA damaging agents used in cancer therapy [9].

Our findings underline some issues that need to be taken into consideration. Firstly, most likely, there are additional germline pathogenic *CHEK2* mutations, not yet discovered. These mutations are likely to be rare and may be distributed across restricted geographical areas. Secondly, such mutations may be of severe clinical importance, e.g. have an impact on cancer risk and sensitivity to chemotherapy [8–10, 16]. Thirdly, as massive parallel sequencing strategies are increasingly being used, not only for research purposes, but also to guide treatment decisions, it is important to note that such germline mutations are likely to be missed by contemporary practice where somatic mutations are being filtered against the germline to remove non-functional SNPs.

## Conclusion

In conclusion, potential germline mutations affecting the *CHEK2* gene should be taken into consideration when exploring the genetic mechanisms of drug resistance among breast and, most likely, also prostatic cancer patients.
